# Optogenetic Application to Investigating Cell Behavior and Neurological Disease

**DOI:** 10.3389/fncel.2022.811493

**Published:** 2022-02-22

**Authors:** Danqing Zhu, Hunter J. Johnson, Jun Chen, David V. Schaffer

**Affiliations:** ^1^California Institute for Quantitative Biosciences, University of California, Berkeley, Berkeley, CA, United States; ^2^Department of Bioengineering, University of California, Berkeley, Berkeley, CA, United States; ^3^Graduate Program in Bioengineering, University of California, San Francisco, San Francisco, CA, United States; ^4^Graduate Program in Bioengineering, University of California, Berkeley, Berkeley, CA, United States; ^5^Department of Molecular and Cell Biology, University of California, Berkeley, Berkeley, CA, United States; ^6^Department of Chemical and Biomolecular Engineering, University of California, Berkeley, Berkeley, CA, United States; ^7^Helen Wills Neuroscience Institute, University of California, Berkeley, Berkeley, CA, United States

**Keywords:** optogenetics, cell signaling, spatiotemporal, gene regulation, neuroscience

## Abstract

Cells reside in a dynamic microenvironment that presents them with regulatory signals that vary in time, space, and amplitude. The cell, in turn, interprets these signals and accordingly initiates downstream processes including cell proliferation, differentiation, migration, and self-organization. Conventional approaches to perturb and investigate signaling pathways (e.g., agonist/antagonist addition, overexpression, silencing, knockouts) are often binary perturbations that do not offer precise control over signaling levels, and/or provide limited spatial or temporal control. In contrast, optogenetics leverages light-sensitive proteins to control cellular signaling dynamics and target gene expression and, by virtue of precise hardware control over illumination, offers the capacity to interrogate how spatiotemporally varying signals modulate gene regulatory networks and cellular behaviors. Recent studies have employed various optogenetic systems in stem cell, embryonic, and somatic cell patterning studies, which have addressed fundamental questions of how cell-cell communication, subcellular protein localization, and signal integration affect cell fate. Other efforts have explored how alteration of signaling dynamics may contribute to neurological diseases and have in the process created physiologically relevant models that could inform new therapeutic strategies. In this review, we focus on emerging applications within the expanding field of optogenetics to study gene regulation, cell signaling, neurodevelopment, and neurological disorders, and we comment on current limitations and future directions for the growth of the field.

## Introduction

Cellular signaling is mediated by highly dynamic and intertwined pathways that control crucial cell behaviors. During organismal development, these signaling processes are spatially and dynamically regulated to orchestrate cell expansion, cell differentiation, and tissue morphogenesis. Likewise, complex signaling pathways enable and protect adult organismal function. Investigating these complex mechanisms has required the development of tools to perturb their activity.

Conventional chemical perturbations can offer some levels of control but have limited capability in their spatial and temporal resolution (Webster et al., 1988; Gossen et al., 1995; Metzger et al., 1995; Rivera et al., 1996; Stanton et al., 2018). As an alternative approach, optogenetics offers the potential for unprecedented precise temporal, spatial, and dosage control of signaling. Specifically, the spatial precision can be achieved on the order of ~micrometers (μm), and the temporal precision can be achieved at ~ms (<1 ms; Shemesh et al., 2017). Optogenetics, which combines genetic and optical tools for the precise perturbation of target cellular processes, initially applied naturally derived photosensitive opsins to study and control the activity of individual neurons and by extension investigate brain circuity. In particular, opsins, derived from various microbial species, were re-purposed for mammalian cell expression and upon illumination at specific wavelengths unleashed ions flow across cell membranes to activate or inhibit target neurons (Zhang et al., 2006; Deisseroth, 2015), as extensively reviewed in recent literature (Looger, 2011; Zhang et al., 2011). This review will focus instead on newly developed light-responsive systems and optics hardware that have enabled applications in new areas such as control of protein activity, signaling dynamics, up- or down- regulation of gene expression, subcellular localization, and other applications. We will discuss how the high spatiotemporal precision of optogenetics has led to novel insights into cellular signaling networks and future trends in the field of optogenetics for a variety of applications in basic science and therapeutics.

## State-Of-The-Art Optogenetic Tools and Optics Technology

**Figure 1:**
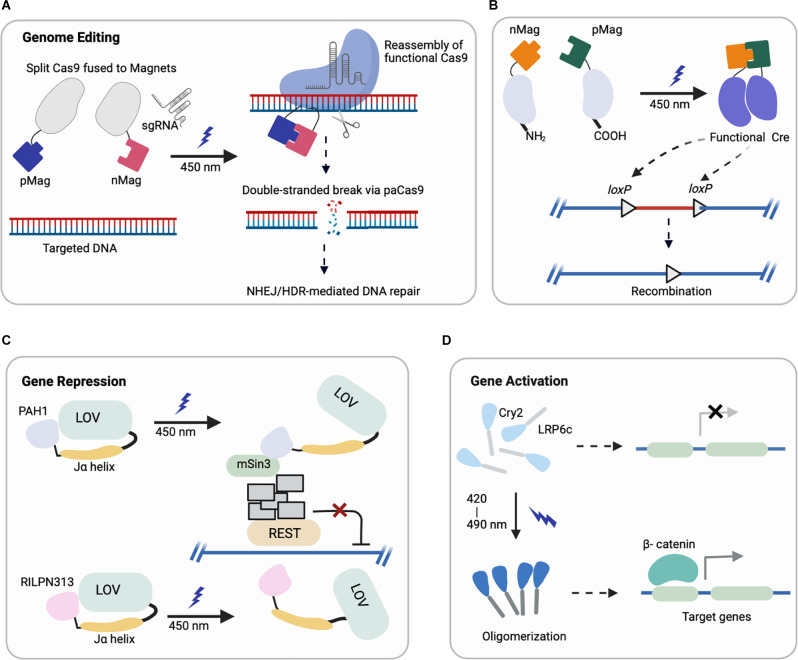
Representative schematics of four major optogenetic-based gene regulations using different systems (created with *Biorender*). **(A,B)** Light-induced uncaging of affinity domains (positive pMag) and (negative nMag) results in dimerization of attached domains and reassembly of **(A)** functional Cas9 for indel mutation and **(B)** split Cre recombinase for DNA recombination. **(C)** Upon light-induction, the J*α* helix unfolds from LOV2 core to uncage a fused protein (PAH1, RILPN313) that inhibits the binding and activity of targeted transcription factor (REST). **(D)** Cry2 clusters an attached protein (LRP6c) in response to light that activates the downstream target genes, e.g., Wnt signaling.

In recent years, optogenetic tools have extended to many classes of proteins containing light-responsive domains (summarized in Table 1) discovered in plants, bacteria, invertebrates, etc. Their photoreceptor domains exhibit light-inducible properties such as reversible protein-protein interactions, oligomerization, and conformational changes, which can be harnessed to engineer photoswitches for regulating gene activation, repression, protein localization, and other functions (Figure 1).

**Table 1:** Summary of common optogenetic systems, mechanisms, and applications.

Systems	Origin	Mechanism	Applications	References
**Cry2/CIB1**	*A. thaliana*	∘ Dimerization ∘ Blue Light (420–490 nm) ∘ Binding of the PHR of photoexcited Cry2 to the CIB1 or CIBN	protein proximity, activation, membrane targeting	Kennedy et al. (2010)
**Cry2 PHR**	*A. thaliana*	∘ Oligomerization/clustering ∘ Blue Light (420–490 nm)	receptor clustering, protein interactions/signaling, subcellular localization	Bugaj et al. (2013)
**AsLOV2**	*A. sativa Phot1*	∘ Caging/conformation change ∘ Blue Light (< 500 nm)	Protein activation, deactivation, subcellular localization	Wu et al. (2009)
**Magnets**	*Neurospora crassa*	∘ Heterodimerization (nMag, pMag) ∘ Small sizes ∘ Fast kinetics	Protein interactions, subcellular localizations, genome editing	Kawano et al. (2015)
**Phy/PIF**	*Arabidopsis thaliana*	∘ Dimerization ∘ Red light (650 nm) activation ∘ Reversed by far-red (750 nm) light ∘ Exogenous chromophore	Deep tissue penetration for protein signaling and interactions control	Levskaya et al. (2009)
**BphP1/PpsR2**	*Rhodopseudomonas palustris*	∘ Dimerization ∘ Near-infrared light (740–780 nm) activation ∘ Chromophore is endogenously present in mammalian cells	Deep tissue penetration for protein signaling and interactions control	Kaberniuk et al. (2016)
**UVR8/COP1**	Plants	∘ Dimerization ∘ UV light (280–315 nm)	Irreversible protein signaling and interactions	Favory et al. (2009)

One important system involves light, oxygen, and voltage domains (LOV), isolated from plant photosensor phototropin 1 (phot1). Phot1 molecule has two LOV domains, with the second domain (LOV2) primarily involved in kinase activation (Christie et al., 1999, 2012; Wu et al., 2009). In particular, *Avena sativa* phot1 LOV2 (AsLOV2) has a tightly associated C-terminal Jα helix in a folded state, but upon exposure to blue light, the bound flavin mononucleotide (FMN) chromophore undergoes a conformational change to displace the Jα helix from the protein core, leading to uncaging of an effector kinase domain (Harper et al., 2003). By engineering fusions between other signaling effectors to LOV domains, this light-induced conformational change can be used to unleash signaling activity.

In another system, cryptochrome 2 (Cry2), derived from the plant *A. thaliana*, has a photolyase homology region (PHR) that interacts with a partner CIB1 in a blue light responsive manner, due to a flavin adenine dinucleotide (FAD) bound to Cry2 as a chromophore cofactor (Kennedy et al., 2010). In one optogenetic design, the Cry2PHR domain can dimerize with CIB1 lacking the basic helix-loop-helix domain (CIBN) within seconds of exposure. In addition to dimerization, Cry2PHR can also be harnessed for self-oligomerization with blue light activation (Bugaj et al., 2013, 2015). Specifically, the detection of protein clusters can be achieved with ~10 s, and dissociation occurs with a half-life ~5.5 min.

In a common system for red light activation, phytochrome (Phy), derived from *Arabidopsis thaliana*, contains two structural domains (Quail, 2010). The N-terminal photosensory domain binds to a tetrapyrrole chromophore, such as phycocyanobilin (PCB), and can be triggered by red light (650 nm) to photoisomerize and bind to phytochrome-interacting factors (PIFs) to modulate downstream pathways (Quail, 2002). This Phy/PIF dimerization has been harnessed to stimulate protein-protein interactions in mammalian cells (Levskaya et al., 2009). More recently, a near-infrared-light (~750 nm) responsive dimerization of bacterial phytochrome (BphP1) with its partner PpsR2 system has reportedly been harnessed for optogenetics (Kaberniuk et al., 2016). In addition to the deep tissue penetration of red- and far-red light, an advantage of Phy/PIF and Bphp1/PpsR2 systems is their reversibility and speed. Specifically, the dissociation of these protein pairs can be induced with lights of different wavelengths, enabling studies to perturb dynamics of signaling networks with a fast on/off rate.

Magnets, an improved system that incorporates positive and negative amino acids into the Vivid (VVD) N-terminal helix, also enables heterodimerization with electrostatic attraction upon uncaging of the protein (Kawano et al., 2015). VVD contains LOV domain that can be excited with blue light illumination to uncage the effector domain (Zoltowski et al., 2007).

Other dimerization systems such as ultraviolet B (UVB) and constitutively photomorphogenic 1 (COP1) offer an alternative excitation wavelength (~280–315 nm). UV response locus 8 (UVR8) can translocate and bind to COP1 in the presence of UVB (Favory et al., 2009), liberating COP1-bound transcription factors in the nucleus to modulate downstream signaling in an irreversible manner (Cloix et al., 2012; Wu et al., 2012).

To activate the variety of photosensory domains that are being harnessed to control signaling pathways and gene expression, the complementary development of optics technologies for illumination is also needed. Current optical stimulation devices have been developed for both *in vitro* and *in vivo* models. In one *in vitro* system, an array of light-emitting diodes (LEDs) can be constructed for large surface illumination in a uniform manner, though spatial resolution can be limited in this design (Tucker et al., 2014). To improve the spatial control of illumination, other systems such as digital micromirror device (DMD) microscopes (Allen, 2017) and light activation at variable amplitudes (LAVA) boards (Repina et al., 2020) have been developed. DMD-based systems offer single-cell resolution with dynamic, user-defined patterns, though with low throughput. By comparison, LAVA boards enable dynamically controlled illumination of multi-well plates for extended culture times, with the option of using masks for spatial patterning with ~100 μm resolution. Other types of devices are summarized in Table 2 (Gerhardt et al., 2016; Bugaj and Lim, 2019). Current *in vivo* hardware primarily relies on optical fiber or implantable LEDs sensors passed through or mounted to the skull of an animal to target cell populations in a spatially restricted manner. Such neural interfaces can generally achieve a temporal precision at ~ ms with a spatial precision ~ hundreds of micrometers (Aravanis et al., 2007; Kim et al., 2013).

**Table 2:** Existing optic hardware used in optogenetic illumination and their characterizations.

	Device Type	Applications	Specifications	References
*In vitro*	Cell culture plate	Whole plate illumination	• Uniform illumination • Large surface area	Tucker et al. (2014)
	Digital micromirror device(DMD)	Microscope illumination	• High temporal resolution (4,000 Hz) • Multiple LEDs • Single cell resolution (5 μm)	Allen (2017)
	LAVA board	24-well/96-well plate illumination	• Single color illumination • Control of light intensity (0–20 μW/mm^2^), time (10 ms resolution), and spatial presentation (100 μm resolution) • GUI control	Repina et al. (2020)
	Light Plate Apparatus (LPA)	24-well plate illumination	• Two color illumination • Control of light intensity (0–3.5 μW/mm^2^) and time (100 ms resolution) • GUI control	Gerhardt et al. (2016)
	optoPlate-96	96-well plate illumination	• Three color illumination • Control of light intensity (0–4 μW/mm^2^) and time (100 ms resolution)	Bugaj and Lim (2019)
*In vivo*	Optic fiber	Laser-coupled optical fiber mounted and passed through the skull	~ hundreds of micrometers ~ milliseconds	Aravanis et al. (2007)
	Wireless optoelectronics	Injectable multimodal sensors with an array microLEDs	~ 50 μm ~ radio frequency scavenging	Kim et al. (2013)

## Optogenetics in Genome Modification and Regulation

Optogenetics has been extensively applied for the spatiotemporal modulation of gene expression, including genome editing, gene activation, and repression.

**(i) Genome Editing**: CRISPR/Cas9 (clustered, regularly interspaced, short palindromic repeats, CRISPR-associated protein 9) can be engineered for targeted genome modification in mammalian cells at specific DNA loci, which enables the causal dissection of gene functions (Cong et al., 2013; Jinek et al., 2013; Mali et al., 2013). Optogenetic control of targeted genome editing can facilitate our improved understanding of gene networks. The first reported photoactivatable Cas9 (paCas9) was based on the dimerization system called Magnets (Kawano et al., 2015). In this work, the Cas9 nucleases were split into two parts fused to photoinducible dimerizing domains (positive Magnet and negative Magnet), which assemble and reconstitute Cas9 activity upon blue light illumination (Nihongaki et al., 2015a). This photoactivatable Cas9 cleaved a targeted endogenous genomic locus and induced indel mutation by nonhomologous end joining (NHEJ) or homology-directed repair (HDR) in a light-dependent fashion (Figure 1A). Using multiple sgRNAs targeting human genes *EMX1* and *VEGFA*, the light-induced Cas9 indel mutations in both genes simultaneously. Though the reported study focused on HEK293T cells, *EMX1* is ubiquitously expressed in neural stem cells and guides differentiation of layer-specific neuronal phenotypes (Schuurmans and Guillemot, 2002), such that this system could be further harnessed for investigating corticogenesis or other neurodevelopmental processes.

Another study adapted a photoactivatable Cre (PA-Cre) system for site-specific genome recombination, where low intensity or short pulsed illumination enabled efficient activation with high spatial and temporal precision *in vivo* (Kawano et al., 2016). In particular, Cre recombinase was split (CreN59/CreC60) into two fragments and fused to components of the Magnet system for heterodimerization under blue light (Figure 1B). This design yielded a substantial increase in DNA recombination compared to a prior design split system (Kennedy et al., 2010). Recently, an improved version of this PA-Cre system was reported to enhance efficiency and reduce dark background recombination when targeting active neurons in the murine dorsal raphe nucleus (Morikawa et al., 2020). Successful recombination was observed under blue light condition for mouse brains *in vivo* and in neural cells *in vitro*, demonstrating the promise of this system for future applications in studying neural pathways in mouse models. Other groups have applied analogous systems with different recombinases to modulate excitatory neurons and parvalbumin interneurons in the mouse brain, which can potentially be combined with PA-Cre for dual locus genome editing (Li et al., 2020).

**(ii) Gene Activation**: Optogenetic control of gene expression has been achieved with several different approaches, and a typical strategy combines a photosensory module with a split transcription factor that can be reassembled upon light illumination. The first reported optogenetic system for regulating gene expression, published in 2002 (Shimizu-Sato et al., 2002), was based on the fusion of the Phy-Gal4-DNA binding domain and a PIF3-Gal4-activation domain to induce downstream gene activation.

Engineering DNA-binding became far easier with the advent CRISPR/Cas9 genome editing, and this capability can be harnessed for other applications. For example, Zhang and colleagues (Konermann et al., 2013) developed light-inducible transcriptional effectors (LITEs) that enable direct optical control of mammalian cell gene expression. Cry2 systems rely upon photoactivated dimerization with its interacting partner CIB1 or the self-oligomerization of the Cry2-PHR. In this study, a customizable DNA-binding domain was fused to the light-sensitive module of Cry2, and upon blue light illumination, its dimerization partner CIB1, fused to a desired effector domain, became recruited to a target locus to modulate gene expression. This approach enabled independent, multiplexed genomic anchors to target a range of genomic loci. More recently, to improve the targeting of multiple sequences in the same gene simultaneously for robust activation, Cry2 was fused to the VP64 transactivation domain while its interacting partner CIB1 was fused to dCas9 (Jinek et al., 2012), the catalytically inactive form of Cas9. With multiple gRNAs co-transfected into mammalian cells to direct the binding of dCas9 to the targeted promoter, transcription could be induced robustly in the presence of blue light. The advantage of such a system compared to the conventional chemical-based strategy in gene activation is reversibility by the removal of light illumination, enabling dynamic patterns of gene modulation (Polstein and Gersbach, 2015). While transcriptional upregulation has been achieved in multiple studies, the magnitude of upregulation can be too low to induce an effective biological response (Nihongaki et al., 2015a, b). To address this shortcoming, a modified system incorporating synergistic activation mediators has been developed (Nihongaki et al., 2017). In this system, a single gRNA is attached to MS2 binding sequences to recruit the MS2 coat protein fused with two activators (p65 and heat shock factor 1) onto the same dCas9 protein, resulting in a 50–100-fold higher activation *(ASCL1)* than the previous constructs in HEK293T cells.

Researchers have applied this system to target *NEUROD1* expression to induce neuronal differentiation in human iPSCs. Using the modified gene activation system, the upregulation was improved by 860-fold after 1 d of blue light illumination, and activated cells stained positively for the neuronal marker β-III tubulin (Tuj1) after 4 d of illumination. Another research has induced functional neuronal differentiation using a far-red light-mediated system, which offers low phototoxicity and deep tissue penetration (Shao et al., 2018).

**(iii) Gene Repression**: In addition to activating target gene expression, multiple systems have been developed to repress transcription. For example, repressive histone modifiers have been fused to light-sensitive proteins to suppress *Grm2* and *Neurog2* gene expression in primary neurons and Neuro2a cells with light illumination (Konermann et al., 2013). Another example harnessed a small peptide degron. Specifically, a small peptide degron RRRGN was fused to the C-terminus of the photoresponsive Jα helix of LOV2 domain. The LOV2 domain contains a flavin-binding core domain, and blue light illumination induces a side-chain rotation of the conserved flavin-interacting residue followed by dissociation and unfolding of the C-terminal Jα helix (Shcherbakova et al., 2015). This dissociation from the LOV core domain under blue light induction exposed the degron, which subsequently stimulated protein degradation (Bonger et al., 2014). Another study further engineered a system to include an additional Cry2-based system together with the LOV-domain, such that the blue light can simultaneously block new mRNA transcription while inducing the degradation of existing proteins (Pathak et al., 2017), showed a further reduction in targeting protein level compared to the single LOV2-system reported previously.

Researchers have also leveraged this LOV2 conformation change to engineer chimeric proteins to inhibit the repressor element (RE) 1-silencing transcription factor (REST) in primary neurons (Paonessa et al., 2016). REST is a transcriptional repressor that is normally expressed at low levels by mature neurons, but in various brain pathologies becomes up regulated (Mandel et al., 2011; Pozzi et al., 2013). A minimal interacting REST sequence was fused to the LOV2 C-terminus. On blue light illumination, LOV2 unfolded and freed the C-terminal domain to interact with and inhibit the binding of REST (Figure 1C). With effective light-medicated REST inhibition, neurons showed increased brain-derived neurotrophic factor transcription and boosted Na^+^ currents and neuronal firing (Paonessa et al., 2016). Future investigation can apply these modulating systems *in vivo* for brain pathologies associated with REST overexpression or hyperactivity (Zuccato et al., 2003; Lu et al., 2014).

Optogenetic tools generally allow modulation of the single gene expression using a single light color system; however, recent studies have highlighted the possibility for multiplexed control of several genes to regulate biological processes (Redchuk et al., 2018), using near infrared (NIR) and blue light controlled systems with minimal spectral crosstalk. In addition, cell lines that include more than one optogenetic system (UVR8, PhyB/PIF, BphP1/PpsR2, etc.) activated by distinct wavelengths can also be generated, enabling downstream exploration of combinatorial or orthogonal signaling pathways (Müller et al., 2014). Other method includes constructing a light-responsive promoter library, which can be induced with pulsatile signaling light and enable multi-gene regulation at a fixed expression ratio (Benzinger and Khammash, 2018).

While these optogenetic tools can modulate gene expressions in targeted cells, recent studies have noted some potential limitations of current systems. These include the chances of toxicity from high intensity blue light illumination, toxicity resulting from high expression levels of Cas activator domain (Ewen-Campen et al., 2017; Casas-Mollano et al., 2020), activity noise, and variation of gene expressions associated with split-component systems (Guinn and Balázsi, 2019). Remedies include using pulses in place of continuous irradiation (Wang et al., 2012), a weaker promoter for Cas constructs (Jia et al., 2018), and negative feedback gene circuit regulation, respectively. As an example of the latter, a recent study demonstrated a five-fold noise reduction by incorporating negative feedback regulation gene circuits (i.e., TetR repressor fused with a Tet-inhibiting peptide; Guinn and Balázsi, 2019). Such synthetic circuits can also be multiplexed orthogonally to probe multigene expressions in contribution of cell phenotypes (Szenk et al., 2020) and buffering gene dosage variation across cell populations for a more homogeneous gene expression control (Yang et al., 2021).

## Optogenetics in Organismal Development

While optogenetics has not yet been broadly applied to study development, there is considerable potential. For example, the nervous system is a highly ordered, complex, and tightly regulated system central to organismal survival and function. This system develops through the expansion and differentiation of various neural stem and progenitor cells, processes that are tightly orchestrated by extracellular ligand signals. These ligands bind to receptor tyrosine kinases (RTKs; Schlessinger, 2014), adhesion, G-protein-coupled (Rosenbaum et al., 2009), and other receptors to initiate signal transduction cascades and trigger downstream cellular responses such as growth, differentiation, migration, and survival. Protein ligands or synthetic agonists have been broadly useful for probing cellular function and malfunction *in vitro* and *in vivo*. However, such signal activation has poor spatial and temporal control, limited by slow diffusion and convection as well as technical limitations in signal presentation and temporal activation. In contrast, optogenetic control of cellular signaling is an exciting approach for spatiotemporal control of signaling (Repina et al., 2017). These approaches thereby offer a valuable toolbox to investigate the complex spatiotemporal role of major signaling pathways in neurodevelopment and adult neurogenesis.

Following gastrulation, precise spatiotemporal presentation of morphogen signals regionally patterns the neural plate, neural groove, and neural tube and thereby the full central nervous system (Nikolopoulou et al., 2017). More specifically, the induction of the neuroectoderm and patterning of both the dorsal-ventral and anterior-posterior axis of the central nervous systems rely on spatiotemporal activation of Wnt, BMP, SHH, FGF, and retinoic acid (RA; Kiecker and Lumsden, 2012). However, challenges with ectopically modulating morphogen signals with temporal and spatial control have classically limited the generation of accurate *in vitro* models of neurodevelopment (Kelava and Lancaster, 2016).

Optogenetic activation of signaling cascades typically relies on a genetically encoded light responsive dimerizing/oligomerizing protein fused to a signaling effector to mimic the effect of upstream ligand binding (Kramer et al., 2021), and it can do so with spatiotemporal control. In particular, several optogenetic systems controlling morphogen signals relevant to development have recently been reported, leveraging Cry2 (Kennedy et al., 2010; Bugaj et al., 2013). One of the first examples of leveraging Cry2 oligomerization to control cellular signaling was optogenetic control of canonical Wnt activation. Our optoWnt system involves fusion of the intracellular portion of the canonical Wnt co-receptor LRP6 to the PHR domain of Cry2 (Figure 1D), and we have reported optogenetic activation of Wnt signaling in both adult neural stem cells (NSCs; Bugaj et al., 2013) and human embryonic stem cells (hESCs; Repina et al., 2020). Other emerging and relevant morphogen signals to neurodevelopment include optoFGFR1(Kim et al., 2014), optoTGF (Li et al., 2018), optoBMP (Humphreys et al., 2020), and optoBrn2 (Sokolik et al., 2015), which have clear applications in modeling neurodevelopment and neurogenesis if translated to either pluripotent stem cells or adult neural stem cells.

optoWnt has been recently deployed to investigate the temporal role of β-catenin activity dynamics on adult neurogenesis (Rosenbloom et al., 2020). It was determined that sustained optoWnt activation promoted proliferation and differentiation, while transient or disrupted activation induced cellular apoptosis. This discrepancy may explain the cell death observed during adult neurogenesis, indicating a potential inherent mechanism that corrects for deviations in differentiation cues.

In studies outside the nervous system, optogenetic control over Ras/ERK signaling in *Drosophila*, relying on the association of Phy-PIF for the membrane localization of SOS proteins (Son of Sevenless), revealed the role of this pathway in promoting endodermal differentiation at the cost of mesodermal fate during a critical time window (McFann et al., 2021). Specifically, Ras signaling was controlled using a specific region of SOS with catalytic activity (SOScat), which activates Ras signal when recruited to the plasma membrane (optoSOS). This was accomplished *via* PIF-SOScat fusion and a membrane PhyB (Toettcher et al., 2013). In hESCs, mixed optoWnt activation in a subpopulation of cells has been linked to mesendoderm specification and migration from a non-Wnt stimulated population, providing insight into gastrulation and early ectoderm specification (Repina et al., 2020). In an alternative application, investigating the temporal dynamics of optoBrn2 in hESC reveals a positive-feedback mechanism, where pluripotency networks do not respond to optoBrn2 signals below a certain magnitude or duration threshold (Sokolik et al., 2015). This result may indicate how hESCs are able to distinguish signal vs. stochastic fluctuations during development. Finally, optogenetics can function as a precision perturbation tool in developmental biology (Krueger et al., 2019). *In vivo* application include decoding signaling dynamics during *Xenopus* development, where light-mediated Raf/MEK/ERK activation, accomplished by membrane localization of Raf1 *via* CRY2-CIBN association, was demonstrated to induce a tail-like structure after germ layer formation (Krishnamurthy et al., 2016, 2017), and optoWnt activation results in axis duplication (Krishnamurthy et al., 2021).

Overall, optogenetic toolkits represent a powerful and exciting approach to understand and probe the mechanisms underlying neurodevelopment and neurogenesis. In the future, optogenetic control of morphogen signals may better inform the spatiotemporal signal dynamics governing neurulation, neural tube patterning, and neural differentiation and enable more physiologically relevant *in vitro* models of these stages.

## Optogenetics in Cellular Function and Synaptic Communication

Beyond early-stage work in neurodevelopment, recent optogenetic systems provide novel approaches to investigate the mechanisms behind cellular survival, function, and synaptic communication in the adult nervous system. One important class of cellular signaling pathways in the nervous system is the tropomyosin-related kinase (Trk) family of RTKs, as they are known to be important for neuronal survival, neurite outgrowth, synaptic function, and synaptic plasticity (Chang et al., 2014). Additionally, Trk signaling cascades are implicated in neurodegenerative disorders, including Alzheimer’s, chronic pain, inflammation, and cancer (Amatu et al., 2019). Upon binding to the respective neurotrophins, members of the Trk family, namely A, B, and C, activate several major signal transduction pathways, including the mitogen-activated protein kinase (MAPK)/extracellular signal-regulated protein kinase (ERK) kinase (MEK), phosphatidylinositol 3-kinase (PI3K)/Akt, and phospholipase Cγ1 (PLCγ1)/Ca^2+^ pathways.

Recently, several such pathways have been placed under light control using Cry2, enabling investigations into mechanisms underlying cellular behavior in the nervous system. For example, an optogenetic TrkA (optoTrkA) system (Duan et al., 2018; Khamo et al., 2019), involving the fusion of the intracellular domain of TrkA to the Cry2-CIB1 dimerizing system, successfully stimulated nerve growth factor (NGF)/TrkA signaling in PC12 cells. Under light stimulation, the group observed both neurite outgrowth and survival of dorsal root ganglion nerves, similar to the effects of exogenous NGF. Another group recently reported that a fusion of the PHR region of Cry2 to the TrkB receptor (optoTrkB) induced robust activation of canonical TrkB signaling (Chang et al., 2014). They demonstrated that sustained light illumination resulted in upregulated extracellular signal related kinase (ERK) activity, neurite outgrowth, and filopodia formation, which mimicked brain-derived neurotrophic factor (BDNF) ligand signaling.

Following similar approaches, Cry2-based optogenetic toolkits for additional signaling cascades have also been recently reported, including optoRaf (Su et al., 2020), optoRhoA (Bugaj et al., 2013), optoCdc42 (Bugaj et al., 2013), optoEphB2 (Locke et al., 2017), and light sensitive myosin motors (Zhang et al., 2021). Overall, these newly developed optogenetic toolkits enable the spatiotemporal profiling of signal activation *in vitro* and enable applications in understanding the signaling dynamics underlying key neurotrophin cascades and cellular function in the nervous system.

In addition to Cry2-based optogenetic systems, the LOV domain from phototropin (Christie et al., 2012) has been harnessed in neurons. For example, in photoactivable Rac1 (Wu et al., 2009) the LOV domain sterically occluded the binding of Rac1 to its effector. Upon light illumination, a conformational change induced unwinding of the Jα helix from the LOV domain and thereby released the inhibition of Rac1 signaling. The researchers thereby demonstrated that selectively activating Rac1 signaling could induce cell motility and control the direction of cell movement, enabling analysis of Rac1 regulation in RhoA in cell mobility. Rac1 is a key GTPase that regulates actin cytoskeletal dynamics (Raftopoulou and Hall, 2004), and in dendritic spines, Rac1 specifically regulates the spine size *via* rearrangement of actin cytoskeleton (Hayashi-Takagi et al., 2010; Yuste, 2011). Previous work has implied the important role of structural plasticity of spines in synaptic transmission for learning-related activities (Hayashi-Takagi et al., 2015). Given the role of Rac1 in neurites and neurons, modulating Rac1 activity using precise and reversible optogenetic tool may offer novel insights into neuronal function and health (Penzes et al., 2011; Hayashi-Takagi, 2017).

Finally, optogenetic control of cellular signaling pathways not only offers exciting new tools to probe biological questions *in vitro*, but it also presents a potential novel perturbation *in vivo* to study mechanisms of neuroregeneration and disease in model organisms. Optogenetic domains have been harnessed for the recruitment of ligands to a specified localization to induce Ca^2+^ signaling *in vivo*. In particular, the PHR domain of Cry2 was fused to STIM1, and light inducible oligomerized STIM1 translocated to the plasma membrane and bound endogenous calcium release-activated calcium (CRAC) channels to trigger Ca^2+^ influx into the cell (Kyung et al., 2015). Using the OptoSTIM1 system, expressed in the hippocampus of living mice, researchers observed an increase of contextual fear memory in the light-illuminated group, suggesting potential mechanisms of Ca^2+^ dependent memory formation. Finally, a recent study demonstrated optoAKT and optoRAF (for the control of ERK signaling) in *Drosophila* enhanced axon regeneration and proper axon guidance in the peripheral nervous system (PNS) as well as axon regrowth and functional recovery in the CNS (Wang et al., 2020). In this example, optogenetics may provide a potential strategy in the intervention of neural degeneration.

Overall, optogenetic control of protein activity is a powerful toolkit to probe the spatiotemporal role of signaling dynamics with high precision and throughput. As new optogenetic systems emerge, future applications in understanding neurodevelopment and function may benefit from *in vitro* and *in vivo* optogenetic approaches.

## Optogenetics in Neurological Disorders and Future Therapeutic Directions

Beyond studying signaling pathways and advancing understanding of complex cellular networks in neurodevelopment and physiological brain functions, optogenetics has also been extended to medical therapeutics and for modulation of cell behaviors in the context of understanding of disease pathologies. The need to develop efficient therapies for neurodegenerative diseases is urgent, given the increasing percentages of the population living longer. One of the reasons for the healthcare burden of neurological disorders is the lack of effective targeted treatments. The simultaneous development of genome engineering and optogenetics has the potential for interrogating mechanistically the pathology involved in genetic-related brain disorders and possibly offering more targeted therapies for clinical applications.

For example, stable, optogenetically-modified mammalian cells could be implanted for relevant clinical conditions (Oggu et al., 2017). Given the deeper tissue penetrance and low toxicity of red or NIR, recently developed red light systems developed for high tissue penetration, transgene, or signal induction with controlled localization, timing, and dosage is increasingly possible in the adult brain. For example, the implantation of wireless, minimally invasive illumination devices can enable optogenetic modulation of neural systems in large organisms (Montgomery et al., 2015; Park et al., 2015). Some previous studies have also shown that light can be efficiently delivered by a helmet-type configuration applied to the brain (Lanzafame et al., 2014). Using engineered stem cells, one can also introduce a therapeutic target gene with a light inducible expression system, which then can be spatiotemporally controlled for producing or inhibiting specific responses (Pomeroy et al., 2017). For example, human embryonic stem cell-mesencephalic dopaminergic neurons have been optogenetically controlled in a model of Parkinson’s disease using a fiber-optic cannula, which exploited optogenetics as an on-off switch for neuronal activity, allowing this function of the cells to be tested independently of other possible functions (Steinbeck et al., 2015). While the study demonstrated the utility of optogenetics to dissect the mechanism underlying graft function, the proof-of-concept of using engrafted, engineered stem cells with optogenetic control is also promising for potential clinical applications of optogenetic cell-based therapies in neurological disorders.

However, there are also some potential limitations in the translation of optogenetic systems to clinical usages, including: (1) the gene cassette size that can be packaged in vectors for transfection; (2) safe and efficient delivery vehicles that can target the cell or tissue of interest (though engineering of viral delivery systems such as engineered adeno-associated viruses can help accelerate progress); (3) potential inflammation and immunogenicity caused by optogenetic tools; (4) limited red-light or NIR light sensitive proteins available with high tissue penetration and low phototoxicity, especially in wavelengths ranges not currently well-explored; (5) potential long-term effects of permanent introduction of non-human proteins with optical properties; and (6) a lack of appropriate illumination devices that present narrower-wavelengths for mitigating surrounding tissue phototoxicity (Wojtovich and Foster, 2014), or high spatial illumination resolution that can circumvent subcellular protein diffusion problems.

To overcome some of these limitations and further improve the light-responsive protein properties, systematic protein engineering processes such as directed evolution (Packer and Liu, 2015; Wu et al., 2019) can be applied to engineer the next generation of optogenetic systems with enhanced efficiency/specificity (Herwig et al., 2017; Bedbrook et al., 2019). Some characteristics to consider can include: (a) specificity in response to an activating wavelength, with low or no background activity under the dark condition, (b) rapid and greater sensitivity to low levels of activation light to allow for longer exposures at low illumination intensity, (c) reduced size of protein components to facilitate delivery.

## Conclusions

In summary, state-of-the-art optogenetic systems are enabling numerous discoveries and insights into fundamental functions of cellular signaling networks. With the substantial acceleration in the range of applications, the role of optogenetics will broadly enable the examination of how genes, proteins, cells, and cellular connections modulate local and global network activity to develop complex tissue structures and encode behaviors. Such fundamental knowledge gained from optogenetic research will also potentially enable clinical therapeutics.

## Author Contributions

DZ and DS substantially contributed to the conception and design of the article and interpreting the relevant literature. DZ wrote the first draft of the manuscript. HJ and JC wrote sections of the manuscript. DS revised the manuscript for important intellectual content. All authors contributed to the article and approved the submitted version.

## Conflict of Interest

The authors declare that the research was conducted in the absence of any commercial or financial relationships that could be construed as a potential conflict of interest.

## Publisher’s Note

All claims expressed in this article are solely those of the authors and do not necessarily represent those of their affiliated organizations, or those of the publisher, the editors and the reviewers. Any product that may be evaluated in this article, or claim that may be made by its manufacturer, is not guaranteed or endorsed by the publisher.
